# Fatty acids in berry lipids of six sea buckthorn (*Hippophae rhamnoides* L., subspecies *carpatica*) cultivars grown in Romania

**DOI:** 10.1186/1752-153X-6-106

**Published:** 2012-09-20

**Authors:** Francisc V Dulf

**Affiliations:** 1 University of Agricultural Sciences and Veterinary Medicine, Cluj-Napoca, Manastur 3-5, 400372, Romania

**Keywords:** Sea buckthorn, *Hippophae rhamnoides* L., Subspecies, Oil content, Fatty acids, Polar lipids, Free fatty acids, Triacylglycerols, Sterol esters, GC-MS

## Abstract

**Background:**

A systematic mapping of the phytochemical composition of different sea buckthorn (*Hippophae rhamnoides* L.) fruit subspecies is still lacking. No data relating to the fatty acid composition of main lipid fractions from the berries of ssp. *carpatica* (Romania) have been previously reported.

**Results:**

The fatty acid composition of the total lipids (oils) and the major lipid fractions (PL, polar lipids; FFA, free fatty acids; TAG, triacylglycerols and SE, sterol esters) of the oils extracted from different parts of six sea buckthorn berry subspecies (ssp. *carpatica*) cultivated in Romania were investigated using the gas chromatography-mass spectrometry (GC-MS). The dominating fatty acids in pulp/peel and whole berry oils were palmitic (23-40%), oleic (20-53%) and palmitoleic (11-27%). In contrast to the pulp oils, seed oils had higher amount of polyunsaturated fatty acids (PUFAs) (65-72%). The fatty acid compositions of TAGs were very close to the compositions of corresponding seed and pulp oils. The major fatty acids in PLs of berry pulp/peel oils were oleic (20-40%), palmitic (17-27%), palmitoleic (10-22%) and linoleic (10%-20%) acids, whereas in seeds PLs, PUFAs prevailed. Comparing with the other lipid fractions the SEs had the highest contents of saturated fatty acids (SFAs). The fatty acid profiles of the FFA fractions were relatively similar to those of TAGs.

**Conclusions:**

All parts of the analyzed sea buckthorn berry cultivars (ssp. *carpatica*) exhibited higher oil content then the other European or Asiatic sea buckthorn subspecies. Moreover, the pulp/peel oils of ssp. *carpatica* were found to contain high levels of oleic acid and slightly lower amounts of linoleic and α-linolenic acids. The studied cultivars of sea buckthorn from Romania have proven to be potential sources of valuable oils.

## Background

Sea buckthorn (SB) (*Hippophae rhamnoides* L. *Elaeagnaceae*) is a bush or a small tree, of the *Elaeagnaceae* family, naturally distributed in Eurasia. The classification of genus *Hippophae* is still unclear. The most common species (sp.), *H. rhamnoides*, was classified in several subspecies (ssp.), including ssp. *carpatica*, which is native in Romania [1]. Over the last decades the SB was domesticated in many countries from Asia, North and South America and Europe, not only for its soil and water conservation ability but also for its yellow-orange berries with an acidic and astringent taste and a high nutritional value. SB berries are rich in a variety of phytochemicals with physiological properties such as vitamins (B, C, E and K), flavonoids, carotenoids, tocopherols and many volatile compounds (i.e., aliphatic esters, alcohols and hydrocarbons [2-4]. Significant amounts of inositols and methylinositols were found in SB berries, which are supposed to contribute to health benefits of SB fruits and derivatives [5]. SB fruit membranes are rich in carotenolipoprotein complexes with 61% phospholipids and 39% galactolipids, as structural components [6]. In vitro and clinical studies show that the SB fruits have positive effect in the treatment of type 1 diabetic patients improving the glucose and lipid metabolism [7], possess high anti-oxidant, hemostatic and anti-inflammatory effects [8,9] and help prevent cardiovascular disease and cancer [10,11].

In last years SB pulp and seed oils have become popular food supplements playing important role in cancer therapy [9]. Several studies have indicated that these berry oils possess important immunostimulant, anti-ulcer and cholesterol-lowering effects, and may also be used in treatment of various skin diseases [12-15].

Both the seeds and soft parts (pulp/peel) of berries show high amounts of oil. The contents of bioactive lipophilic compounds, (i.e., phytosterols (up to 23 g/kg in seed oil and up to 29 g/kg in pulp/peel oil), tocopherols and tocotrienols (up to 2.9 g/kg in seed oil and up to 1.8 g/kg in pulp oil) and carotenoids (up to 3.5 g/kg in pulp oil) are generally high in the extracted seed and pulp/peel oils [2,16,17]. The existing studies reported different chemical compositions for SB seed and pulp/peel oils which vary widely depending on the subspecies, harvesting time of the fruits and the many other climatic and geographical conditions. Whereas the seed oil contains high amounts of unsaturated fatty acids, with linoleic (C18:2*n*-6) (30-40%) and α-linolenic (C18:3*n*-3) (20-35%) acid as the dominating fatty acids, the pulp/peel oil is rich in palmitoleic (C16:1*n*-7) (16-54%) and palmitic acids (C16:0) (17-47%) being more saturated [16,18,19]. The TAGs and PLs are the major lipid fractions in both of SB seed and pulp/peel oils [17].

A systematic mapping of the phytochemical composition of different SB fruits subspecies is still lacking. Ssp. *carpatica* is the most cultivated sea buckthorn ssp. in Romania. No data relating to the fatty acid composition of main lipid fractions from this berry ssp. have been previously reported. The purpose of the present study was to characterize the fatty acid composition of the total lipids (oils) and the major lipid fractions (PLs, FFAs, TAGs and SEs) of the oils extracted from different fruit parts of six SB subspecies (ssp. *carpatica*) cultivated in Romania.

## Results and discussion

### Oil content of the SB materials

**Figure 1:**
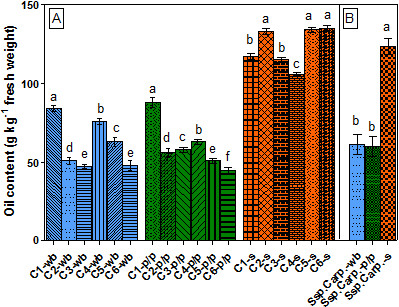
**Oil content (g kg**^**-1**^**fresh weight) of sea buckthorn berries (ssp. *carpatica*): A- oil content of different parts of six cultivars; B- the average oil content in analyzed parts of berries (mean of six cultivars).**

The oil content of seeds, soft parts and whole berries (based on fresh weight, f.w.) of different SB cultivars (ssp. *carpatica*) are presented in Figure 1-A. The oil amounts of the analyzed berry parts varied widely: 45–84 g kg ^-1^- in whole berries, 45- 88 g kg ^-1^- in pulp/peel and 106–135 g kg ^-1^- in seeds. The average oil content in seeds of the studied SB ssp. (123 g kg ^-1^) was significantly higher (p < 0.05) than in soft parts (60 g kg ^-1^) and whole berries (62 g kg ^-1^), respectively (Figure 1-B). These results are similar with the oil contents of ssp. *mongolica*, and higher than those reported for ssp. *sinensis* (97 g kg ^-1^ seeds, f.w. and 41 g kg ^-1^ berry, f.w.) [16]. Yang et al. [17] determined the following amounts of oils for ssp. *rhamnoides*: 11% (f.w.) in seeds, 3% (f.w.) in soft parts and 3.5% (f.w.) in whole berries, respectively. Gutierrez et al. [18] concluded that the drying methods of SB berry parts could affect the oil extraction yield. These authors reported significant differences between the total oil content of air-dried berry pulp (cultivar *Indian-summer*) and freeze-dried pulp (36% vs. 16% (weight/weight, w/w)), whereas the total lipid recovery from air-dried seeds and freeze-dried seeds were similar (11% and 12% (w/w)).

### Fatty acid composition in oil of pulp/peel, seeds and whole berries

The fatty acid compositions of pulp/peel, seeds and whole berries oils of six SB berry cultivars (ssp. *carpatica*) are listed in Tables 1 and 2. Due to the dominance of the pulp and peels in SB fruit, the composition of the oil from the whole berry resembled that of the pulp/peel oil.

**Table 1:** **Fatty acid composition (weight % of total fatty acids) of oils from whole berries, pulp/peel and seeds of different cultivars of *H. rhamnoides *L. (ssp. *carpatica*) fruits**

	**Sea buckthorn cultivars (ssp. *carpatica*)**					
**Fatty acid**	**C1**	**C2**	**C3**	**C4**	**C5**	**C6**
			**Whole berries**			
C14:0	0.22 ± 0.05	0.61 ± 0.10	0.59 ± 0.10	0.25 ± 0.05	0.37 ± 0.03	0.33 ± 0.05
C15:0	tr	0.05 ± 0.02	0.04 ± 0.02	0.03 ± 0.01	0.03 ± 0.02	0.04 ± 0.01
C16:0	35.11 ± 0.80	20.80 ± 0.61	36.16 ± 0.84	37.33 ± 0.87	37.21 ± 0.89	33.32 ± 0.64
C16:1n-9	0.02 ± 0.01	0.14 ± 0.02	0.04 ± 0.02	0.02 ± 0.01	0.02 ± 0.01	0.03 ± 0.02
C16:1n-7	19.80 ± 0.55	9.63 ± 0.38	24.64 ± 0.46	23.70 ± 0.65	23.75 ± 0.75	19.65 ± 0.60
C17:0	0.03 ± 0.02	0.03 ± 0.02	0.02 ± 0.01	0.02 ± 0.01	0.02 ± 0.01	tr
C18:0	1.41 ± 0.17	2.86 ± 0.14	0.94 ± 0.10	0.96 ± 0.12	0.82 ± 0.08	1.27 ± 0.10
C18:1n-9	30.47 ± 0.73	45.90 ± 0.80	22.29 ± 0.62	23.93 ± 0.73	24.85 ± 0.65	28.39 ± 0.91
C18:1n-7	6.78 ± 0.20	4.55 ± 0.30	6.23 ± 0.20	6.58 ± 0.22	5.76 ± 0.22	5.37 ± 0.17
C18:2n-6	3.05 ± 0.13	10.87 ± 0.38	6.24 ± 0.25	5.17 ± 0.20	4.57 ± 0.23	7.60 ± 0.25
C18:3n-3	2.90 ± 0.14	4.17 ± 0.15	2.67 ± 0.13	1.86 ± 0.14	2.41 ± 0.19	3.86 ± 0.16
C20:0	0.17 ± 0.05	0.23 ± 0.04	0.12 ± 0.02	0.12 ± 0.03	0.14 ± 0.03	0.13 ± 0.03
C20:1n-9	0.06 ± 0.02	0.15 ± 0.02	0.03 ± 0.01	0.02 ± 0.01	0.05 ± 0.02	tr
			**Pulp/peel**			
C14:0	0.23 ± 0.03	0.59 ± 0.06	0.46 ± 0.04	0.29 ± 0.05	0.42 ± 0.05	0.40 ± 0.04
C15:0	tr	0.04 ± 0.02	0.03 ± 0.02	0.03 ± 0.02	0.03 ± 0.01	0.04 ± 0.02
C16:0	34.62 ± 0.88	23.17 ± 0.63	39.11 ± 0.91	38.76 ± 1.11	39.22 ± 1.22	37.68 ± 1.12
C16:1n-9	0.04 ± 0.02	0.16 ± 0.04	0.02 ± 0.01	0.01 ± 0.01	0.02 ± 0.01	0.03 ± 0.02
C16:1n-7	19.53 ± 0.67	11.05 ± 0.44	26.70 ± 0.58	25.74 ± 0.96	26.19 ± 0.71	24.90 ± 0.90
C17:0	0.03 ± 0.02	0.02 ± 0.01	0.02 ± 0.01	0.02 ± 0.01	0.03 ± 0.02	0.02 ± 0.01
C18:0	1.25 ± 0.15	2.53 ± 0.07	0.84 ± 0.06	0.77 ± 0.08	0.61 ± 0.07	0.87 ± 0.08
C18:1n-9	32.76 ± 0.94	53.08 ± 1.12	20.81 ± 0.69	22.75 ± 0.75	24.41 ± 0.74	23.10 ± 0.82
C18:1n-7	6.41 ± 0.29	5.34 ± 0.16	6.41 ± 0.20	6.85 ± 0.25	5.72 ± 0.18	6.31 ± 0.19
C18:2n-6	4.06 ± 0.16	2.25 ± 0.10	4.57 ± 0.18	4.15 ± 0.16	2.57 ± 0.09	3.41 ± 0.10
C18:3n-3	0.84 ± 0.08	1.33 ± 0.07	0.90 ± 0.05	0.54 ± 0.04	0.63 ± 0.04	3.14 ± 0.11
C20:0	0.17 ± 0.03	0.24 ± 0.04	0.10 ± 0.02	0.07 ± 0.02	0.12 ± 0.03	0.10 ± 0.03
C20:1n-9	0.06 ± 0.03	0.20 ± 0.05	0.03 ± 0.01	tr	0.05 ± 0.02	tr
			**Seeds**			
C14:0	0.10 ± 0.02	0.09 ± 0.03	0.24 ± 0.03	0.15 ± 0.03	0.12 ± 0.02	0.09 ± 0.01
C15:0	0.11 ± 0.03	tr	0.30 ± 0.04	tr	0.12 ± 0.03	tr
C16:0	9.12 ± 0.38	7.14 ± 0.26	12.44 ± 0.44	9.43 ± 0.33	10.29 ± 0.31	8.06 ± 0.28
C16:1n-9	nd	nd	nd	nd	nd	nd
C16:1n-7	0.53 ± 0.07	0.16 ± 0.03	0.36 ± 0.03	0.43 ± 0.06	0.33 ± 0.04	0.19 ± 0.03
C17:0	0.03 ± 0.01	0.03 ± 0.02	tr	0.05 ± 0.01	0.03 ± 0.01	tr
C18:0	3.03 ± 0.07	3.84 ± 0.08	2.91 ± 0.09	3.68 ± 0.11	3.10 ± 0.10	2.98 ± 0.08
C18:1n-9	13.57 ± 0.53	14.89 ± 0.41	16.74 ± 0.66	15.49 ± 0.51	16.30 ± 0.60	20.09 ± 0.71
C18:1n-7	2.28 ± 0.11	1.38 ± 0.08	1.48 ± 0.10	2.22 ± 0.10	2.29 ± 0.11	1.27 ± 0.07
C18:2n-6	42.35 ± 0.95	42.12 ± 1.13	33.72 ± 0.98	36.98 ± 0.82	34.41 ± 1.04	38.93 ± 1.17
C18:3n-3	28.50 ± 0.55	29.78 ± 0.62	31.81 ± 0.72	30.98 ± 0.70	32.60 ± 0.80	28.13 ± 0.67
C20:0	0.37 ± 0.04	0.41 ± 0.04	0.21 ± 0.04	0.49 ± 0.03	0.35 ± 0.04	0.26 ± 0.04
C20:1n-9	tr	0.16 ± 0.03	tr	0.10 ± 0.02	0.06 ± 0.02	tr

Values are mean *±* SD of three samples of each fruit part, analyzed individually in triplicate; C1- C6, sea buckthorn (ssp. *carpatica*) cultivars.

C14:0, myristic; C15:0, pentadecanoic; C16:0, palmitic; C16:1n-9, *cis*-7 hexadecenoic; C16:1n-7, palmitoleic; C17:0, margaric; C18:0, stearic; C18:1n-9, oleic; C18:1n-7, vaccenic; C18:2n-6, linoleic; C18:3n-3, α-linolenic; C20:0, arachidic; C20:1n-9, eicosenoic acids.

**Table 2:** **Fatty acid composition (weight % of total fatty acids) of oils from different parts of sea buckthorn fruits (ssp. *carpatica*)**

	**Sea buckthorn cultivars (ssp. *carpatica*)**					
**Fatty acid classes**	**C1**	**C2**	**C3**	**C4**	**C5**	**C6**
			Whole berries			
∑ SFAs	36.94 ± 1.09_b_^ab^	24.58 ± 0.93_b_^c^	37.87 ± 1.09_b_^ab^	38.72 ± 1.09_b_^a^	38.59 ± 1.06_b_^a^	35.09 ± 0.83_b_^b^
∑ MUFAs	57.12 ± 1.51_a_^ab^	60.37 ± 1.52_a_^a^	53.22 ± 1.31_a_^c^	54.26 ± 1.62_a_^bc^	54.43 ± 1.65_a_^bc^	53.45 ± 1.70_a_^c^
∑ PUFAs	5.95 ± 0.27_c_^e^	15.05 ± 0.53_c_^a^	8.91 ± 0.38_c_^c^	7.03 ± 0.34_c_^d^	6.98 ± 0.42_c_^de^	11.46 ± 0.41_c_^b^
*PUFAs/SFAs*	*0.16*^d^	*0.61*^a^	*0.24*^c^	*0.18*^cd^	*0.18*^cd^	*0.33*^b^
*n–6/n–3*	*1.05*^e^	*2.61*^b^	*2.34*^c^	*2.79*^a^	*1.90*^d^	*1.97*^d^
			Pulp/peel			
∑ SFAs	36.30 ± 1.11_b_^b^	26.59 ± 0.83_b_^c^	40.56 ± 1.06_b_^a^	39.95 ± 1.29_b_^a^	40.41 ± 1.40_b_^a^	39.11 ± 1.30_b_^ab^
∑ MUFAs	58.80 ± 1.95_a_^b^	69.83 ± 1.81_a_^a^	53.96 ± 1.49_a_^b^	55.36 ± 1.97_a_^b^	56.39 ± 1.66_a_^b^	54.34 ± 1.93_a_^b^
∑ PUFAs	4.90 ± 0.24_c_^c^	3.58 ± 0.17_c_^d^	5.47 ± 0.23_c_^b^	4.69 ± 0.20_c_^c^	3.20 ± 0.13_c_^d^	6.56 ± 0.21_c_^a^
*PUFAs/SFAs*	*0.13*^ab^	*0.13*^ab^	*0.13*^ab^	*0.12*^bc^	*0.08*^c^	*0.17*^a^
*n–6/n–3*	*4.83*^b^	*1.70*^d^	*5.05*^b^	*7.67*^a^	*4.11*^c^	*1.09*^e^
			Seed			
∑ SFAs	12.77 ± 0.55_c_^bc^	11.51 ± 0.43_c_^c^	15.89 ± 0.64_c_^a^	13.79 ± 0.51_c_^b^	14.00 ± 0.51_c_^b^	11.39 ± 0.41_c_^c^
∑ MUFAs	16.38 ± 0.71_b_^d^	16.59 ± 0.55_b_^cd^	18.58 ± 0.79_b_^b^	18.24 ± 0.69_b_^bc^	18.99 ± 0.77_b_^b^	21.55 ± 0.81_b_^a^
∑ PUFAs	70.84 ± 1.50_a_^ab^	71.90 ± 1.75_a_^a^	65.53 ± 1.70_a_^c^	67.97 ± 1.52_a_^abc^	67.01 ± 1.84_a_^bc^	67.06 ± 1.84_a_^bc^
*PUFAs/SFAs*	*5.55*^c^	*6.25*^a^	*4.12*^e^	*4.93*^d^	*4.79*^d^	*5.89*^b^
*n–6/n–3*	*1.49*^a^	*1.41*^b^	*1.06*^e^	*1.19*^d^	*1.06*^e^	*1.38*^c^

C1- C6, sea buckthorn (ssp. *carpatica*) cultivars.

Values are mean *±* SD of three samples of each fruit part, analyzed individually in triplicate. Means in the same row followed by different superscript letters indicate significant differences (p *<* 0.05) among cultivars (C1-C5); means in the same column followed by different subscript letters indicate significant differences (p *<* 0.05) between fatty acid classes of each cultivar; SFAs, saturated fatty acids; MUFAs, monounsaturated fatty acids; PUFAs, polyunsaturated fatty acids.

The fatty acid levels of the seed and berry pulp/peel oil varied widely.

The dominating fatty acids in berry pulp/peel oils were palmitic (16:0) (23-40%), oleic (18:1n-9) (20-53%) and palmitoleic (16:1n-7) (11-27%). Small or trace amounts of vaccenic (18:1n-7), linoleic(18:2n-6), α-linolenic (18:3n-3), stearic (18:0), myristic (14:0), pentadecanoic (15:0), *cis*-7 hexadecenoic (16:1n-9), margaric (17:0) and two long chain fatty acids, arachidic (20:0) and eicosenoic (20:1n-9) acids were observed in all analyzed soft part oils. In two cultivars, C1 and C2, the proportions of oleic acid (32.76% for C1 and 53.08% for C2) exceeded that of the palmitoleic acid (19.53% for C1 and 11.05% for C2). From these results can be concluded that MUFAs were the dominant fatty acid classes (53-70%), followed by SFAs (26-41%) and PUFAs (3-7%) (Table 2). The PUFA/SFA ratios were close to zero, with a significantly high value (0.17) (p < 0.05) in pulp/peel oil of C6. Statistically significant differences (p < 0.05) were observed between n-6/n-3 ratios of analyzed berry pulp/peel oils, with the highest value in cultivar C4 (7.67) and the lowest in C6 (1.09), respectively (Table 2).

Similar amounts of palmitic (in cv. *Indian-summer* and *H. rhamnoides* (India)), vaccenic (in cv. *Indian-summer* and ssp. *sinensis*) and α-linolenic (in cv. *Indian-summer*, *H. rhamnoides* (India) and *H*. *salcifolia*) acids were recently reported by different authors for berry pulp oil. Higher proportions of palmitoleic acid and much lower levels of oleic acid were characteristic of the Finnish, Chinese and Canadian soft part SB oils, excepting species *H. tibetana* which presented similar percentages of (18:1n-9) with those of results from the present study [2,17,18].

Seed oils consisted mainly of linoleic, α-linolenic, oleic, palmitic and stearic acids, with minor or trace amounts of vaccenic, palmitoleic, arachidic, eicosenoic, myristic, pentadecanoic and margaric acids (Table 1). A notable feature of the berry seed oils was the extremely low level of palmitoleic acid (0.1-0.5%). The relatively high deviations were observed in the proportions of oleic (13-21%) and linoleic (33-43%) acids. In contrast to the pulp oils, seed oils had higher amounts of PUFAs (65-72%) and lower proportions of MUFAs (16–21.5%) and SFAs (11-16%), respectively (Table 2). These oils, characterized by high ratios of PUFAs/SFAs, with an extremely significant high value (p < 0.05) for cultivar C2 (6.25), are susceptible to oxidative damage due to their high α-linolenic acid content (28-33%). Statistically significant variations (p < 0.05) were observed between n-6/n-3 ratios of analysed six seed oils, with all the values close to 1 (Table 2). This phenomenon could be explained by the ratio of linoleic to α-linolenic acid (close to 1:1), which is different from the main vegetable oils [20,21]. Generally the proportions of unsaturated fatty acids from seed oils obtained in this study were in accordance with those reported by Yang and Kallio [17] and Yang et al. [22] for ssp. *sinensis* and *rhamnoides*. The concentration of α-linolenic was found slightly higher in air- and freeze- dried SB seed oils (~ 37% and ~ 39%, respectively) of cv. *Indian-summer* than in the corresponding oils from the present work [18].

The high amount of palmitoleic acid, unusual for a vegetable oil, distinguishes the berry pulp/peel oils from the seed oils of SB. This valuable fatty acid, which is an important component of skin fat, has attracted an increasing interest due to its hypocholesterolemic and hypoglyceridemic activities [2].

**Figure 2:**
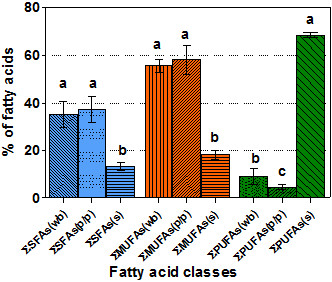
**Comparison of the fatty acid classes compositions (as % of total fatty acids) from the oils of different parts of sea buckthorn fruits (ssp. *carpatica*).**

Comparing the average proportions (average of six cultivars) of the fatty acid classes from the oils of different parts of berries, the seed oil contained significantly lower proportions of SFAs and MUFAs (p < 0.05), and significantly higher amount of PUFAs (p < 0.05), than the whole berry and pulp/peel oils (Figure 2).

### Fatty acid composition in individual lipid fractions of oils from pulp/peel and seeds

The fatty acid compositions of the main lipid classes (PLs, FFAs, TAGs and SEs) from pulp/peel and seed oils are presented in Tables 3, 4, 5 and 6.

**Table 3:** **Fatty acid composition (weight % of total fatty acids) of individual lipid classes from pulp/peel oils of different cultivars (C1-C6) of sea buckthorn fruits (ssp. *carpatica*)**

**Fatty acids (weight % of total fatty acids; mean *± *SD, n = 3)**													
**Species**	**C14:0**	**C15:0**	**C16:0**	**C16:1n-9**	**C16:1n-7**	**C17:0**	**C18:0**	**C18:1n-9**	**C18:1n-7**	**C18:2n-6**	**C18:3n-3**	**C20:0**	**C20:1n-9**
**C1**													
PL	0.36 ± 0.03	nd	24.48 ± 0.82	nd	14.57 ± 0.42	nd	1.34 ± 0.04	34.13 ± 0.85	6.21 ± 0.20	10.82 ± 0.45	7.54 ± 0.25	0.55 ± 0.04	nd
FFA	1.25 ± 0.10	nd	32.09 ± 0.80	nd	17.70 ± 0.48	nd	18.20 ± 0.60	18.80 ± 0.57	3.94 ± 0.11	5.82 ± 0.22	2.20 ± 0.10	nd	nd
TAG	0.26 ± 0.04	0.03 ± 0.02	38.98 ± 1.10	0.12 ± 0.03	21.16 ± 0.52	0.06 ± 0.02	0.98 ± 0.10	28.98 ± 0.75	6.13 ± 0.25	2.69 ± 0.11	0.49 ± 0.04	0.11 ± 0.02	0.02 ± 0.01
SE	1.35 ± 0.12	nd	27.53 ± 0.90	0.90 ± 0.04	1.52 ± 0.09	0.52 ± 0.06	38.85 ± 1.11	20.24 ± 0.56	0.27 ± 0.05	6.19 ± 0.25	0.94 ± 0.04	1.70 ± 0.08	nd
**C2**													
PL	0.34 ± 0.04	nd	17.52 ± 0.58	nd	10.34 ± 0.38	nd	1.13 ± 0.04	39.57 ± 0.80	5.18 ± 0.19	17.09 ± 0.60	8.83 ± 0.28	tr	nd
FFA	1.50 ± 0.10	nd	33.98 ± 0.89	nd	14.83 ± 0.42	nd	17.26 ± 0.58	22.55 ± 0.40	3.60 ± 0.14	4.60 ± 0.18	1.68 ± 0.06	nd	nd
TAG	0.57 ± 0.04	0.02 ± 0.01	24.39 ± 0.78	0.32 ± 0.03	13.81 ± 0.46	tr	2.04 ± 0.12	48.83 ± 0.90	5.75 ± 0.18	2.52 ± 0.10	1.48 ± 0.12	0.13 ± 0.02	0.15 ± 0.02
SE	1.65 ± 0.09	nd	27.77 ± 0.63	0.60 ± 0.04	1.12 ± 0.11	0.42 ± 0.04	36.52 ± 0.84	22.82 ± 0.78	0.32 ± 0.04	6.60 ± 0.28	0.68 ± 0.10	1.50 ± 0.07	nd
**C3**													
PL	0.55 ± 0.05	nd	23.97 ± 0.48	nd	21.00 ± 0.58	nd	1.40 ± 0.15	20.55 ± 0.55	7.71 ± 0.30	19.45 ± 0.70	4.72 ± 0.18	0.64 ± 0.04	nd
FFA	1.32 ± 0.08	nd	35.52 ± 0.95	nd	16.20 ± 0.62	nd	18.20 ± 0.53	18.84 ± 0.50	3.20 ± 0.12	4.82 ± 0.15	1.90 ± 0.06	nd	nd
TAG	0.38 ± 0.04	tr	40.16 ± 1.18	0.08 ± 0.02	26.31 ± 0.72	0.04 ± 0.02	0.83 ± 0.06	19.81 ± 0.40	6.70 ± 0.15	4.84 ± 0.14	0.63 ± 0.07	0.16 ± 0.02	0.08 ± 0.02
SE	1.42 ± 0.08	nd	26.53 ± 0.52	0.82 ± 0.03	1.42 ± 0.19	0.50 ± 0.05	39.89 ± 1.15	16.60 ± 0.42	0.82 ± 0.06	8.60 ± 0.28	1.20 ± 0.05	2.20 ± 0.10	nd
**C4**													
PL	0.72 ± 0.05	nd	27.22 ± 0.72	nd	19.90 ± 0.58	nd	0.88 ± 0.05	23.24 ± 0.62	8.08 ± 0.25	14.74 ± 0.48	4.75 ± 0.16	0.48 ± 0.04	nd
FFA	1.88 ± 0.08	nd	36.42 ± 0.80	nd	16.72 ± 0.68	nd	16.85 ± 0.62	18.15 ± 0.45	3.12 ± 0.13	4.38 ± 0.16	2.48 ± 0.12	nd	nd
TAG	0.28 ± 0.04	tr	40.45 ± 1.12	0.03 ± 0.02	25.64 ± 0.72	0.02 ± 0.01	0.82 ± 0.04	21.17 ± 0.43	7.03 ± 0.28	3.96 ± 0.15	0.46 ± 0.04	0.14 ± 0.02	tr
SE	0.88 ± 0.06	nd	29.22 ± 0.72	0.30 ± 0.04	0.90 ± 0.08	0.20 ± 0.04	33.18 ± 0.72	25.44 ± 0.70	1.20 ± 0.05	5.20 ± 0.20	1.60 ± 0.05	1.88 ± 0.09	nd
**C5**													
PL	0.26 ± 0.03	nd	20.27 ± 0.57	nd	22.12 ± 0.80	nd	3.48 ± 0.14	27.22 ± 0.60	7.31 ± 0.25	14.21 ± 0.32	4.60 ± 0.20	0.52 ± 0.03	nd
FFA	1.72 ± 0.10	nd	30.54 ± 0.81	nd	14.65 ± 0.46	nd	16.90 ± 0.65	23.70 ± 0.82	4.20 ± 0.16	5.89 ± 0.19	2.40 ± 0.10	nd	nd
TAG	0.30 ± 0.04	tr	39.19 ± 0.91	0.06 ± 0.02	24.20 ± 0.52	0.02 ± 0.01	0.94 ± 0.10	24.94 ± 0.71	6.53 ± 0.22	2.93 ± 0.11	0.62 ± 0.06	0.20 ± 0.03	0.08 ± 0.02
SE	2.20 ± 0.09	nd	26.42 ± 0.52	0.70 ± 0.03	1.40 ± 0.11	0.70 ± 0.04	40.05 ± 0.92	19.60 ± 0.54	0.48 ± 0.06	5.80 ± 0.20	1.00 ± 0.05	1.65 ± 0.05	nd
**C6**													
PL	0.45 ± 0.05	nd	21.75 ± 0.57	nd	22.07 ± 0.60	nd	1.21 ± 0.09	25.83 ± 0.75	7.33 ± 0.30	15.10 ± 0.30	5.91 ± 0.19	0.36 ± 0.03	nd
FFA	1.68 ± 0.08	nd	33.09 ± 0.61	nd	15.60 ± 0.42	nd	15.64 ± 0.45	21.78 ± 0.48	4.10 ± 0.15	5.95 ± 0.15	2.17 ± 0.10	nd	nd
TAG	0.42 ± 0.04	0.05 ± 0.02	36.97 ± 1.13	0.08 ± 0.03	25.59 ± 0.92	0.03 ± 0.01	0.96 ± 0.09	24.82 ± 0.65	6.66 ± 0.28	3.53 ± 0.14	0.68 ± 0.05	0.13 ± 0.02	0.10 ± 0.03
SE	1.20 ± 0.06	nd	24.20 ± 0.61	1.10 ± 0.03	1.30 ± 0.12	0.88 ± 0.03	39.80 ± 0.88	22.34 ± 0.66	0.82 ± 0.05	5.70 ± 0.18	0.78 ± 0.07	1.88 ± 0.07	nd

PL- polar lipids, FFA- free fatty acids, TAG- triacylglycerols, SE- sterol esters.

**Table 4:** **Fatty acid composition (weight % of total fatty acids) of individual lipid classes from seed oils of different cultivars (C1-C6) of sea buckthorn fruits (ssp. *carpatica*)**

**Fatty acids (weight % of total fatty acids; mean *± *SD, n = 3)**													
**Species**	**C14:0**	**C15:0**	**C16:0**	**C16:1n-9**	**C16:1n-7**	**C17:0**	**C18:0**	**C18:1n-9**	**C18:1n-7**	**C18:2n-6**	**C18:3n-3**	**C20:0**	**C20:1n-9**
**C1**													
PL	0.16 ± 0.02	0.13 ± 0.03	17.21 ± 0.64	nd	0.26 ± 0.04	tr	6.30 ± 0.20	14.23 ± 0.57	4.32 ± 0.15	45.48 ± 1.22	11.09 ± 0.45	0.82 ± 0.04	tr
FFA	0.46 ± 0.04	tr	25.33 ± 0.80	nd	0.41 ± 0.02	tr	9.13 ± 0.28	17.98 ± 0.62	4.56 ± 0.14	30.34 ± 0.90	11.79 ± 0.40	tr	nd
TAG	0.09 ± 0.02	0.14 ± 0.02	8.19 ± 0.25	nd	0.55 ± 0.02	0.04 ± 0.02	2.51 ± 0.16	17.94 ± 0.66	2.27 ± 0.09	43.65 ± 1.12	24.22 ± 0.82	0.29 ± 0.03	0.10 ± 0.02
SE	0.56 ± 0.05	0.05 ± 0.02	24.59 ± 0.62	nd	0.22 ± 0.03	0.39 ± 0.04	29.36 ± 0.77	13.37 ± 0.43	1.78 ± 0.08	18.05 ± 0.50	7.94 ± 0.22	3.68 ± 012	nd
**C2**													
PL	0.06 ± 0.02	0.06 ± 0.03	16.33 ± 0.42	nd	0.09 ± 0.02	tr	6.93 ± 0.28	14.56 ± 0.40	3.41 ± 0.15	46.98 ± 1.23	10.31 ± 0.38	0.96 ± 0.04	0.30 ± 0.03
FFA	1.20 ± 0.08	0.30 ± 0.02	26.32 ± 0.62	nd	0.20 ± 0.03	0.30 ± 0.04	11.20 ± 0.38	16.20 ± 0.48	3.10 ± 0.12	27.78 ± 0.85	12.20 ± 0.40	1.20 ± 0.05	nd
TAG	0.06 ± 0.03	0.09 ± 0.02	5.63 ± 0.18	nd	0.16 ± 0.03	tr	2.32 ± 0.16	13.56 ± 0.54	1.25 ± 0.05	44.02 ± 1.10	32.68 ± 0.95	0.12 ± 0.03	0.12 ± 0.03
SE	0.25 ± 0.03	0.02 ± 0.01	26.20 ± 0.82	nd	0.30 ± 0.04	0.42 ± 0.05	28.40 ± 0.72	12.27 ± 0.50	1.65 ± 0.06	17.55 ± 0.68	8.74 ± 0.30	4.20 ± 0.18	nd
**C3**													
PL	0.15 ± 0.02	0.10 ± 0.03	18.69 ± 0.52	nd	0.15 ± 0.02	0.14 ± 0.03	8.64 ± 0.32	12.72 ± 0.52	4.05 ± 0.16	40.90 ± 0.95	13.33 ± 0.42	0.99 ± 0.06	0.15 ± 0.2
FFA	1.60 ± 0.08	0.20 ± 0.02	25.80 ± 0.76	nd	0.30 ± 0.03	0.60 ± 0.06	15.10 ± 0.44	15.20 ± 0.39	1.98 ± 0.05	25.80 ± 0.85	12.52 ± 0.52	0.90 ± 0.07	nd
TAG	tr	tr	7.99 ± 0.28	nd	0.19 ± 0.02	tr	3.55 ± 0.20	17.72 ± 0.68	1.84 ± 0.06	36.05 ± 1.10	31.77 ± 0.88	0.60 ± 0.05	0.28 ± 0.04
SE	0.62 ± 0.03	0.04 ± 0.02	25.20 ± 0.78	nd	0.28 ± 0.03	0.26 ± 0.03	31.68 ± 0.88	10.82 ± 0.40	1.42 ± 0.06	16.80 ± 0.65	7.28 ± 0.28	5.60 ± 0.20	nd
**C4**													
PL	0.12 ± 0.03	0.06 ± 0.02	17.29 ± 0.50	nd	0.21 ± 0.03	0.11 ± 0.02	6.95 ± 0.25	12.61 ± 0.38	4.62 ± 0.17	43.08 ± 1.20	13.85 ± 0.52	1.10 ± 0.06	tr
FFA	1.10 ± 0.06	0.15 ± 0.03	27.58 ± 0.60	nd	0.15 ± 0.02	0.25 ± 0.05	14.80 ± 0.38	12.85 ± 0.42	2.85 ± 0.10	27.10 ± 0.90	12.20 ± 0.40	0.97 ± 0.07	nd
TAG	0.06 ± 0.02	0.12 ± 0.03	8.76 ± 0.30	nd	0.41 ± 0.04	0.04 ± 0.02	3.10 ± 0.10	15.55 ± 0.55	2.28 ± 0.12	36.84 ± 1.18	32.26 ± 0.80	0.47 ± 0.06	0.11 ± 0.03
SE	0.42 ± 0.03	0.02 ± 0.01	27.20 ± 0.72	nd	0.18 ± 0.02	0.35 ± 0.04	30.13 ± 1.00	11.25 ± 0.32	1.60 ± 0.07	16.15 ± 0.60	6.90 ± 0.20	5.80 ± 0.15	nd
**C5**													
PL	0.11 ± 0.02	tr	20.62 ± 0.80	nd	0.09 ± 0.03	0.10 ± 0.02	7.16 ± 0.22	12.33 ± 0.52	4.46 ± 0.20	40.86 ± 1.25	13.04 ± 0.38	1.22 ± 0.08	tr
FFA	0.93 ± 0.04	0.23 ± 0.03	20.09 ± 0.78	nd	tr	tr	12.26 ± 0.40	13.37 ± 0.44	2.96 ± 0.12	29.53 ± 1.00	19.55 ± 0.62	1.09 ± 0.06	nd
TAG	0.07 ± 0.03	tr	8.82 ± 0.32	nd	0.41 ± 0.05	0.02 ± 0.01	2.73 ± 0.09	15.75 ± 0.50	2.40 ± 0.10	35.28 ± 1.10	34.03 ± 1.12	0.41 ± 0.04	0.07 ± 0.02
SE	0.50 ± 0.03	tr	23.80 ± 0.84	nd	0.20 ± 0.04	0.30 ± 0.04	32.80 ± 0.98	12.30 ± 0.48	1.90 ± 0.06	15.85 ± 0.45	6.90 ± 0.25	5.45 ± 0.20	nd
**C6**													
PL	0.10 ± 0.02	0.08 ± 0.02	20.23 ± 0.54	nd	0.14 ± 0.03	0.23 ± 0.03	6.94 ± 0.18	15.30 ± 0.39	3.91 ± 0.16	41.54 ± 1.22	10.51 ± 0.42	1.02 ± 0.05	tr
FFA	1.44 ± 0.06	0.28 ± 0.04	24.64 ± 0.78	nd	0.41 ± 0.06	0.50 ± 0.05	14.41 ± 0.38	14.01 ± 0.39	2.67 ± 0.12	26.12 ± 0.95	14.00 ± 0.60	1.53 ± 0.06	nd
TAG	0.05 ± 0.02	0.12 ± 0.03	7.24 ± 0.30	nd	0.29 ± 0.03	0.05 ± 0.01	2.86 ± 0.12	18.61 ± 0.52	1.77 ± 0.07	39.70 ± 1.12	28.85 ± 0.90	0.31 ± 0.03	0.16 ± 0.03
SE	0.30 ± 0.04	tr	25.20 ± 0.82	nd	0.28 ± 0.03	0.40 ± 0.06	31.80 ± 1.10	12.80 ± 0.42	1.30 ± 0.06	17.20 ± 0.50	5.80 ± 0.20	4.92 ± 0.18	nd

PL- polar lipids, FFA- free fatty acids, TAG- triacylglycerols, SE- sterol esters.

**Table 5:** **Fatty acid composition (weight % of total fatty acids) of individual lipid classes from pulp/peel oils of different cultivars of sea buckthorn fruits (ssp.*carpatica*)**

	**Fatty acids (weight % of total fatty acids)**				
**Species**	**∑ SFA**	**∑ MUFA**	**∑ PUFA**	**PUFA/ SFA**	**n-6/ n-3**
C1					
PL	26.73 ± 0.93_d_^b^	54.91 ± 1.47_a_^a^	18.36 ± 0.70_a_^c^	0.69_a_	1.44_d_
FFA	51.54 ± 1.50_b_^a^	40.44 ± 1.16_b_^b^	8.02 ± 0.32_b_^c^	0.16_b_	2.65_c_
TAG	40.41 ± 1.30_c_^b^	56.41 ± 1.56_a_^a^	3.18 ± 0.15_c_^c^	0.08_b_	5.49_b_
SE	69.94 ± 2.27_a_^a^	22.93 ± 0.74_c_^b^	7.13 ± 0.29_b_^c^	0.10_b_	6.59_a_
C2					
PL	18.99 ± 0.66_d_^c^	55.09 ± 1.37_b_^a^	25.92 ± 0.88_a_^b^	1.36_a_	1.93_c_
FFA	52.74 ± 1.57_b_^a^	40.98 ± 0.96_c_^b^	6.28 ± 0.24_b_^c^	0.12_b_	2.74_b_
TAG	27.15 ± 0.97_c_^b^	68.86 ± 1.59_a_^a^	3.99 ± 0.22_c_^c^	0.15_b_	1.71_c_
SE	67.86 ± 1.67_a_^a^	24.86 ± 0.97_d_^b^	7.28 ± 0.38_b_^c^	0.11_b_	9.71_a_
C3					
PL	26.56 ± 0.72_d_^b^	49.26 ± 1.43_b_^a^	24.18 ± 0.88_a_^c^	0.91_a_	4.12_c_
FFA	55.04 ± 1.56_b_^a^	38.24 ± 1.24_c_^b^	6.72 ± 0.21_c_^c^	0.12_b_	2.54_d_
TAG	41.56 ± 1.32_c_^b^	52.97 ± 1.31_a_^a^	5.47 ± 0.21_c_^c^	0.13_b_	7.67_a_
SE	70.54 ± 1.90_a_^a^	19.66 ± 0.70_d_^b^	9.80 ± 0.33_b_^c^	0.14_b_	7.17_b_
C4					
PL	29.29 ± 0.86_d_^b^	51.21 ± 1.45_a_^a^	19.49 ± 0.64_a_^c^	0.67_a_	3.10_b_
FFA	55.15 ± 1.50_b_^a^	37.99 ± 1.26_b_^b^	6.86 ± 0.28_b_^c^	0.12_b_	1.77_c_
TAG	41.70 ± 1.23_c_^b^	53.87 ± 1.45_a_^a^	4.43 ± 0.19_c_^c^	0.11_b_	8.52_a_
SE	65.36 ± 1.63_a_^a^	27.84 ± 0.87_c_^b^	6.80 ± 0.25_b_^c^	0.10_b_	3.25_b_
C5					
PL	24.53 ± 0.77_d_^b^	56.65 ± 1.65_a_^a^	18.82 ± 0.52_a_^c^	0.77_a_	3.09_c_
FFA	49.16 ± 1.56_b_^a^	42.55 ± 1.44_b_^b^	8.29 ± 0.29_b_^c^	0.17_b_	2.45_d_
TAG	40.64 ± 1.09_c_^b^	55.81 ± 1.42_a_^a^	3.55 ± 0.17_d_^c^	0.09_c_	4.77_b_
SE	71.02 ± 1.62_a_^a^	22.18 ± 0.74_c_^b^	6.80 ± 0.25_c_^c^	0.10_c_	5.80_a_
C6					
PL	23.77 ± 0.74_d_^b^	55.22 ± 1.65_a_^a^	21.01 ± 0.49_a_^c^	0.88_a_	2.56_c_
FFA	50.41 ± 1.14_b_^a^	41.47 ± 1.05_b_^b^	8.12 ± 0.25_b_^c^	0.16_b_	2.75_c_
TAG	38.55 ± 1.31_c_^b^	57.25 ± 1.91_a_^a^	4.20 ± 0.19_d_^c^	0.11_c_	5.22_b_
SE	67.96 ± 1.65_a_^a^	25.56 ± 0.86_c_^b^	6.48 ± 0.25_c_^c^	0.10_c_	7.31_a_

Values are mean *±* SD of three samples, analyzed individually in triplicate

Means in the same row followed by different superscript letters indicate significant differences (p *<* 0.05) among fatty acid classes; means in the same column followed by different subscript letters indicate significant differences (p *<* 0.05) among lipid classes of each cultivar.

*PL*, polar lipids; *FFA*, free fatty acids; *TAG*, triacylglycerols; *SE*, steryl esters; *SFA*, saturated fatty acids; *MUFA*, monounsaturated fatty acids; *PUFA*, polyunsaturated fatty acids.

**Table 6:** **Fatty acid composition (weight % of total fatty acids) of individual lipid classes from seed oils of different cultivars of sea buckthorn fruits (ssp.*carpatica*)**

	**Fatty acids (weight % of total fatty acids)**				
**Species**	**∑ SFA**	**∑ MUFA**	**∑ PUFA**	**PUFA/ SFA**	**n-6/ n-3**
**C1**					
PL	24.62 ± 0.93_c_^b^	18.81 ± 0.76_b_^c^	56.57 ± 1.67_b_^a^	2.30_b_	4.10_a_
FFA	34.92 ± 1.12_b_^b^	22.95 ± 0.78_a_^c^	42.13 ± 1.30_c_^a^	1.21_c_	2.57_b_
TAG	11.26 ± 0.50_d_^c^	20.87 ± 0.79_ab_^b^	67.87 ± 1.94_a_^a^	6.03_a_	1.80_d_
SE	58.63 ± 1.62_a_^a^	15.37 ± 0.54_c_^c^	25.99 ± 0.72_d_^b^	0.44_d_	2.27_c_
**C2**					
PL	24.34 ± 0.79_c_^b^	18.36 ± 0.60_a_^c^	57.29 ± 1.61_b_^a^	2.35_b_	4.56_a_
FFA	40.52 ± 1.19_b_^a^	19.50 ± 0.63_a_^b^	39.98 ± 1.25_c_^a^	0.99_c_	2.28_b_
TAG	8.22 ± 0.42_d_^c^	15.08 ± 0.65_b_^b^	76.70 ± 2.05_a_^a^	9.33_a_	1.35_d_
SE	59.49 ± 1.81_a_^a^	14.22 ± 0.60_b_^c^	26.29 ± 0.98_d_^b^	0.44_d_	2.01_c_
**C3**					
PL	28.70 ± 0.98_c_^b^	17.07 ± 0.72_b_^c^	54.23 ± 1.37_b_^a^	1.89_b_	3.07_a_
FFA	44.20 ± 1.43_b_^a^	17.48 ± 0.47_b_^c^	38.32 ± 1.37_c_^b^	0.87_c_	2.06_c_
TAG	12.14 ± 0.53_d_^c^	20.03 ± 0.80_a_^b^	67.83 ± 1.98_a_^a^	5.59_a_	1.13_d_
SE	63.40 ± 1.94_a_^a^	12.52 ± 0.49_c_^c^	24.08 ± 0.93_d_^b^	0.38_d_	2.31_b_
**C4**					
PL	25.63 ± 0.88_c_^b^	17.44 ± 0.58_ab_^c^	56.93 ± 1.72_b_^a^	2.22_b_	3.11_a_
FFA	44.85 ± 1.19_b_^a^	15.85 ± 0.54_b_^c^	39.30 ± 1.30_c_^b^	0.88_c_	2.22_b_
TAG	12.55 ± 0.53_d_^c^	18.35 ± 0.74_a_^b^	69.10 ± 1.98_a_^a^	5.51_a_	1.14_c_
SE	63.92 ± 1.95_a_^a^	13.03 ± 0.41_c_^c^	23.05 ± 0.80_d_^b^	0.36_d_	2.34_b_
**C5**					
PL	29.22 ± 1.14_c_^b^	16.88 ± 0.75_ab_^c^	53.90 ± 1.63_b_^a^	1.84_b_	3.13_a_
FFA	34.60 ± 1.31_b_^b^	16.32 ± 0.56_b_^c^	49.08 ± 1.62_c_^a^	1.42_c_	1.51_c_
TAG	12.05 ± 0.49_d_^c^	18.63 ± 0.67_a_^b^	69.32 ± 2.22_a_^a^	5.75_a_	1.04_d_
SE	62.85 ± 2.09_a_^a^	14.40 ± 0.58_c_^c^	22.75 ± 0.70_d_^b^	0.36_d_	2.30_b_
**C6**					
PL	28.60 ± 0.84_c_^b^	19.35 ± 0.58_a_^c^	52.05 ± 1.64_b_^a^	1.82_b_	3.95_a_
FFA	42.80 ± 1.37_b_^a^	17.08 ± 0.57_b_^c^	40.12 ± 1.55_c_^b^	0.94_c_	1.87_c_
TAG	10.62 ± 0.51_d_^c^	20.83 ± 0.65_a_^b^	68.55 ± 2.02_a_^a^	6.46_a_	1.38_d_
SE	62.62 ± 2.20_a_^a^	14.38 ± 0.51_c_^c^	23.00 ± 0.70_d_^b^	0.37_d_	2.97_b_

Values are mean *±* SD of three samples, analyzed individually in triplicate

Means in the same row followed by different superscript letters indicate significant differences (p *<* 0.05) among fatty acid classes; means in the same column followed by different subscript letters indicate significant differences (p *<* 0.05) among lipid classes of each cultivar.

*PL*, polar lipids; *FFA*, free fatty acids; *TAG*, triacylglycerols; *SE*, steryl esters; *SFA*, saturated fatty acids; *MUFA*, monounsaturated fatty acids; *PUFA*, polyunsaturated fatty acids.

#### Fatty acid composition of TAGs

**Figure 3:**
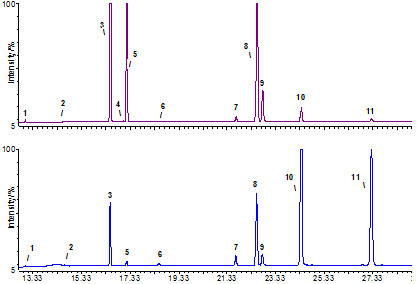
**GC-MS chromatogram of FAMEs from the TAGs of pulp/peel (a) and seeds (b) of sea buckthorn berries (ssp. *carpatica*).**

**Figure 4:**
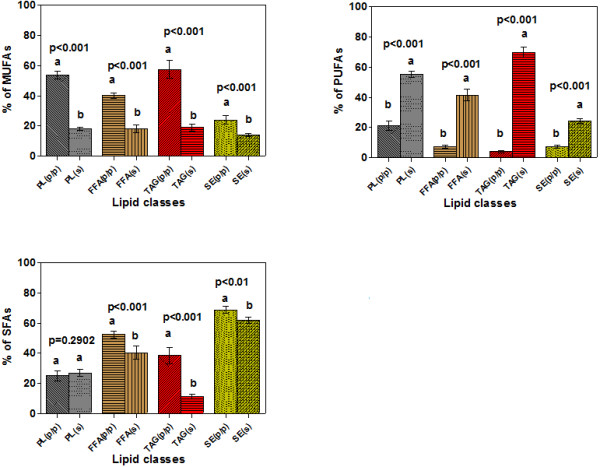
**The average proportions of fatty acid classes (3a- % of MUFAs, 3b- % of SFAs, 3c- % of PUFAs) in lipid fractions from pulp/peel and seeds of sea buckthorn berries (ssp. *carpatica*).**

The fatty acid compositions of TAGs (Figure 3) were very close to the compositions of corresponding seed and pulp oils, with the same dominating fatty acid classes (Table 1; Figure 4 (a), (b) and (c)).

#### Fatty acid composition of PLs

The dominating fatty acids in descending order in berry pulp/peel oils were oleic (20-40%), palmitic (17-27%), palmitoleic (10-22%), linoleic (10%-20%) and α-linolenic (4-9%) acids (Table 3). In all PL fractions extremely significant differences (p < 0.05), were observed between the proportions of fatty acid classes, with the MUFAs as the major fatty acids (Table 5). All the values of PUFA/SFA ratios were close to 1, varying between 0.67 (for C4) and 1.36 (for C2), respectively. Comparing the pulp/peel lipid fractions from the studied cultivars, PLs presented the highest values (p < 0.05) for PUFA/SFA ratios. The n-6/n-3 ratios varied between 1.4 (in C1) and 4.1 (in C3) (Table 5). Recent studies have shown that a balanced intake of dietary PUFA and SFA (ranged between 1.0 and 1.5) can contribute to reduce cardiovascular diseases [23,24]. The glycerophospholipids from pulp/peel oils of subspecies *sinensis*, *rhamnoides* and *mongolica* presented greater amounts of the 18:2n-6 (25.7%, 24.2% and 32.1%, respectively) and 18:3n-3 (15.4%, 12.9% and 10%, respectively) fatty acids than those of corresponding PLs from the present study [16,17]. The phospholipid fractions from SB pulp oils of cv. *Indian- summer* exhibited much higher amounts of palmitoleic (22.7-25%) and lower amounts of oleic (1.4-2.4%) acids than coresponding samples of this work [18].

In seeds PLs, PUFAs were present in a significantly greater proportion (p < 0.05), than SFAs and MUFAs (Tables 4 and 6). The oleic and linoleic acid contents (Table 4) were comparable with the values reported for the seeds of Asian and European SB berries [16-18]. Small variations of n-6/n-3 ratios were observed for the seed oils PLs, the values (Table 6) being close to the recommended essential fatty acid balance, reported in literature [25]. As shown in Figure 4 (a) and (c) the average value of MUFAs was significantly higher, in the berry pulp/peel oil PL than in the seed oil PL (53.5% vs 17.9%, p < 0.001) and vice versa for PUFAs (21.3% vs 54.9%, p < 0.001).

#### Fatty acid composition of SEs

The major fatty acids in ascending order in all berry soft part oils were linoleic (5-9%), oleic (16-26%), palmitic (24-30%), and stearic (33-41%). The relatively high values of n-6/n-3 ratios of the berry pulp/peel oils SEs closely resembled those of the berry pulp/peel oil TAGs, excepting cultivars C2 and C4 (see Table 5). Comparing with the other lipid fractions from these oils, the SEs had the highest content of SFAs (p < 0.05). This class of fatty acids were also predominant in seed oil SEs due to their high content of palmitic and stearic acids (Tables 4 and 6).

It is interesting to note that the arachidic acid levels were around of 2% in pulp/peel oils SEs and between 3% and 6% in seed oils SEs.

The long chain saturated fatty acids, with more than 20 carbons, are major structural components of plant cuticular lipids [26].

Average proportions of MUFAs and SFAs were significantly higher in pulp/peel oils SEs than in seed oils SEs (p < 0.01) and vice versa for PUFAs (p < 0.001) (see Figure 4 (a), (b) and (c)).

The levels of SFAs from studied SB oils SEs were comparable to those reported for other berry SE fractions [27,28].

#### Fatty acid composition of FFA

The fatty acid profiles of the FFA fractions of pulp/peel and seed oils were relatively similar to those of TAGs excepting the proportions for stearic acid (in berry pulp/peel oils) and for palmitic, stearic and α-linolenic acids (in seed oils), respectively (Tables 3 and 4). Generally, the SFAs were the most representative in all analysed cultivars, followed by MUFAs in pulp/peel and PUFAs in seed oils FFAs, respectively (Tables 5 and 6). Low levels of free fatty acids (2-4%) have been reported for oils from air- and freeze- dried SB (cultivar *Indian- Summer*) seeds and pulps by Gutierrez et al. [18], with the similar fatty acid profiles to those of neutral lipids. The quality of the vegetable oils depends on their lipid profile. A high proportion of the free fatty acids offers an unacceptable flavour to the oils [29]. Differences between the fatty acid profiles of the studied lipid fractions could be due to the different phases of biosynthesis and accumulation of TAGs, SEs, PLs and fatty acids. In the first stage PLs and SEs are synthesized with the SFAs as dominating fatty acid classes in their composition. The TAGs proportion, with high unsaturated fatty acid content, increases in the second phase of biosynthesis [28,30,31].

## Conclusions

This study provides valuable information about the fatty acid composition of the major lipid fractions (PLs, FFAs, TAGs and SEs) in the oils extracted from different berry parts of six SB subspecies (ssp. *carpatica*).

Comparing with the other European or Asiatic SB subspecies, all berry parts of the analyzed cultivars exhibited higher oil content. Moreover, the pulp/peel oils of ssp. *carpatica* were found to contain high levels of oleic acid and slightly lower amounts of linoleic and α-linolenic acids.

The PLs presented the highest PUFA/SFA ratios between the analysed pulp/peel lipid fractions (from 0.67 to 1.36), values which were close to the recommended PUFA/SFA intake of nutrition scientists (1–1.5).

The seed oils could be considered excellent sources of PUFAs due to their high contents of linoleic and α-linolenic acids which in human body are precursors of other long-chain n-3 and n-6 fatty acids.

The data obtained in the present work are useful to identify suitable SB cultivars when organizing the berry breeding programs and also provides important information for food and pharmaceutical industry.

## Methods

### Samples and chemicals

Berries of SB (*Hippophae rhamnoides* L., ssp. *carpatica*, cvs. Auras (C1), Serpenta (C2), Tiberiu (C3), Victoria (C4), Ovidiu (C5) and Silvia (C6)) were collected from the experimental field of the Fruit Research Station- Bacau, Romania. The fruits were collected during June to November of 2011 at the stage of commercial maturity and were stored in polyethylene bags at -20°C until analysis.

Seeds were isolated manually from the fruits just before analysis at the laboratory.

Standards of fatty acid methyl esters (37component FAME Mix, SUPELCO, catalog No: 47885-U) were purchased from Supelco (Bellefonte, PA, USA). All reagents, chemicals of analytical or HPLC purity and polar lipid standards were purchased from Sigma–Aldrich (St. Louis, MO, USA). The thin layer chromatography (TLC) plates (silica gel 60 F_254_, 20 × 20 cm) were purchased from Merck (Darmstadt, Germany).

### Lipid extraction

The oils of the whole berries, pulp/peel and seeds were extracted from 5 g of samples using a methanol/chloroform extraction procedure [17,32]. The sample was homogenized in methanol (50 mL) for 1 min with a high-power homogeniser (MICCRA D-9, Germany), chloroform (100 mL) was added, and homogenization was continued for a further 2 min. The mixture was filtered and the solid residue resuspended in chloroform: methanol (2:1, v/v, 150 mL) and homogenized for another 3 min. The mixture was filtered again and washed with 150 mL chloroform: methanol (2:1, v/v). The filtrates were combined and cleaned with 0.88% potassium chloride water solution and methanol: water (1:1, v/v) solution. The bottom layer containing the purified lipids was filtered before the solvent was removed on a rotary evaporator. The lipid samples were transferred to vials with 4 mL chloroform (stock solution), and stored at −18°C until they were analyzed.

### Fatty acid composition

Fatty acid methyl esters (FAMEs) were obtained from lipids using acid-catalysed transesterification procedure described by Christie [33].

For total FAME analysis, 0.2 mL of each oil extract (stock solution) was dissolved in 1 ml toluene and then methylated with 1% sulfuric acid in methanol (2 ml), using a 15 mL screw-cap Pyrex culture tube at 80°Cfor 2 h. After cooling to room temperature, 5 ml of water (with 5%NaCl) and 2 mL hexane were added. The hexane layer was collected and concentrated before the FAMEs were applied to TLC plates. The loaded TLC plates were developed in a mixture of petroleum ether: diethyl ether: acetic acid (85:15:1, v/v/v), sprayed with 2’, 7’-dichlorofluoroscein/methanol (0.1% w/v) and viewed under UV light (254 nm) [34]. The corresponding FAME band was scraped and eluted with chloroform. The eluent was removed with a gentle nitrogen stream. The FAMEs were dissolved in 1 mL hexane and placed into a gas chromatography (GC) vial. The vial was capped and placed at −18°C until GC analysis.

The lipid classes (PLs, FFAs, TAGs and SEs) were separated also by TLC. For fractionation, 0.2 ml of each oil (stock solution) was applied on the TLC plates, developed and viewed under UV light as above. The polar lipids remained at the origin of the plates (the first band). The other major lipid class bands from TLC plates, were identified using commercial standards (which were run in parallel with the samples) and then scraped from the plates. The bands for PLs and FFAs were eluted with methanol: chloroform (1:1, v/v), and the upper two major bands corresponding to TAGs and SE respectively, were eluted with chloroform. After the chloroform was evaporated under a nitrogen stream, the lipid classes were methylated (20 min at reflux for PLs and 2 h at reflux for the other lipid fractions). The extraction of the corresponding FAMEs in hexane was done as described above.

### Analysis of FAMEs by GC

The FAMEs were determined by gas chromatography–mass spectrometry (GC-MS), using a PerkinElmer Clarus 600 T GC-MS (PerkinElmer, Inc., Shelton, U.S.A.) equipped with a SUPELCOWAX 10 column (60 m × 0.25 mm i.d., 0.25 μm film thickness; Supelco Inc., Bellefonte, PA). The initial oven temperature was 140°C, increased to 220°C with a rate of 7°C/min and then held at this temperature for 23 min. Flow rate of the carrier gas He and the split ratio were 0.8 ml/min and 1:24, respectively. The injector temperature was 210°C. The positive ion electron impact (EI) mass spectra was recorded at an ionization energy of 70 eV and a trap current of 100 μA with a source temperature of 150°C. The mass scans were performed within the range of m/z: 22–395 at a rate of 0.14 scan/s with an intermediate time of 0.02 s between the scans. The injection volume was 0.5 μl. Identification of FAMEs was done comparing their retention times with those of known standards (37component FAME Mix, SUPELCO # 47885-U) and the resulting mass spectra to the ones from our database (NIST MS Search 2.0).

### Statistical analyses

All the extractions and GC-MS analysis were made in triplicate. Dates were expressed as mean ± S.D. Statistical differences among samples were estimated using Student’s *t-*test and ANOVA (Tukey’s Multiple Comparison Test; GraphPad Prism Version 4.0, Graph Pad Software Inc., San Diego CA). P < 0.05 was accepted to be statistical significant.

## Abbreviations

Ssp: Subspecies; sp: Species; PLs: Polar lipids; FFAs: Free fatty acids; TAGs: Triacylglycerols; SEs: Sterol esters; PUFAs: Polyunsaturated fatty acids; SFAs: Saturated fatty acids; MUFAs: Monounsaturated fatty acids; SB: Sea buckthorn; f.w.: Fresh weight; w/w: Weight/weight; cv: Cultivars; FAMEs: Fatty acid methyl esters; TLC: Thin layer chromatography; GC-MS: Gas chromatography–mass spectrometry.

## Competing interests

The author declares that he has no competing interests.

## Authors’ contributions

FVD carried out all experiments and prepared the final manuscript.
